# The Damage to Thick Steel Plates by Local Contact Explosions

**DOI:** 10.3390/ma16082966

**Published:** 2023-04-08

**Authors:** Yanghua He, Zhenyi Liu, Mingzhi Li, Pengliang Li, Yao Zhao, Qiqi Liu, Chuang Liu, Ping Ye

**Affiliations:** State Key Laboratory of Explosion Science and Technology, Beijing Institute of Technology, Beijing 100081, China

**Keywords:** explosive, local contact explosion, steel plate, damage mode, numerical simulation

## Abstract

The paper presents the damage results of thick steel plates subjected to local blast loading using experimental and numerical approaches. Three steel plates with a thickness of 17 mm under the local contact explosion of trinitrotoluene (TNT) explosives were tested, and the damaged parts of the steel plates were scanned using a scanning electron microscope (SEM). ANSYS LS-DYNA software was used to simulate the damage results of the steel plate. By analyzing and comparing the experimental results with the numerical simulation results, the influence law of the TNT acting on the steel plate, the damage mode of the steel plate, the reliability verification of the numerical simulation, and the criterion for judging the damage mode of the steel plate were obtained. Results show that the damage mode of the steel plate changes with the changes in the explosive charge. The diameter of the crater on the surface of the steel plate is mainly related to the diameter of the contact surface between the explosive and the steel plate. The fracture mode of the steel plate in the process of generating cracks is a quasi-cleavage fracture, and the process of generating craters and perforations in the steel plate is a ductile fracture. The damage mode of the steel plates can be divided into three types. The numerical simulation results have minor errors and high reliability, and numerical simulation can be used as an auxiliary tool for experiments. A new criterion is proposed to predict the damage mode of the steel plates under contact explosion.

## 1. Introduction

Explosions will cause large damage to steel structures. The study of low-cost small-sized steel plates can provide a reliable basis for the optimal design of ships, pipelines, military equipment, and high-security buildings containing steel structures [1,2]. Research in this area started with experimental investigation and theoretical analysis a long time ago [3,4,5]. With the development of computer technology and detection technology, new experimental studies based on SEM [6] and numerical simulation studies based on experimental studies [7] have been added.

In the experimental research, the main factors affecting the damage mode of the steel plate are the magnitude of the impact load, the loading method, and the fixing method and properties of the steel plate [5]. In the research on the effect of the impact load on steel plates, Nurick [8] investigated the maximum permanent displacement of steel plates by different explosive shapes during contact explosion. Curry [9,10] studied the effect of the amount of explosives and the distance between the explosive and the steel plate on the permanent deformation of the steel plate. In the study of the influence of the properties of the steel plate on the damage mode of the steel plate, McDonald [11,12] evaluated the deformation resistance and rupture threshold of four types of high-strength steel plates by explosive long-range explosion experiments, and a new proximity loading parameter that can predict the deformation of the steel plate was proposed. Zhang [13], Elveli [14], and Li [15] studied the effect of prefabrication defects on the deformation and damage of steel plates under blast impact through experiments and numerical simulations. Hou [16] and Wang [17] studied the effect of coatings with different properties on the impact load of the steel plate and obtained the damage mode of the steel plate under the protection of the layer. Song [18] obtained four damage modes for the pipeline steel when the explosives contacted and exploded through the experimental study of the thickness of 14.6 mm and 26.2 mm steel pipes with different explosives. Li [19,20] demonstrated that the spall strength of low carbon steel under impact is mainly determined by peak stress and strain rate, and the influence of pulse duration is small. 

Based on the experimental study of the SEM, the fracture reason, fracture location, and fracture mode of a steel plate can be obtained. Geffroy [21] found that two types of damage caused by voids and micro-cracks occurred during the deformation process of plate fracture. Wang [22] found that the interface between γ-austenite and B2 phase will fracture first in the dynamic fracture process of high specific strength steel under impact. According to Amerii [23], the quasi-cleavage fracture is the primary fracture mode of steel plate spallation in the process of impact.

The dynamic response and final deformation of plates are the main directions of theoretical research. Fu [2] revised the model for predicting the final deformation of the armor plate under the blast load using theoretical research on the steel plate strain rate and the geometric scaling ratio on the specific impulse correction factor, combined with the relevant experimental study and numerical simulation research. Yao [24] proposed a new dimensionless number of steel plates under the action of air explosion and underwater explosion through dimensionless analysis and obtained a dynamic response prediction formula for plates under different load explosion conditions. By integrating previous experiments and numerical simulation results, Lomazzi [25] proposed a modeling framework for predicting the permanent lateral deflection of an initial quadrilateral slab under blast loading.

Feasibility verification and predictions for steel plate deformation are the main contents of numerical simulation research. Compared with theoretical analysis, the prediction results of numerical simulations are more visualized. Aune [26] studied the dynamic response of the thin steel plate under blast load using the fluid–structure interaction method. By comparing the experimental data, it was confirmed that fluid–structure interaction is feasible for simulating the deflection and velocity of the thin steel plate. Sumelka [27] studied the dynamic damage of an aluminum plate under an air explosion load using numerical simulation and verified the applicability of numerical simulations by comparing it with the experimental results. Mehreganian [28] proved that the coupled method is more consistent with the behavior of the plate after the explosion by comparing the numerical simulation with the experiment. In terms of the finite element algorithm, most scholars [18,29] adopt the fluid–structure coupling algorithm, Lagrangian mesh is used for the main damage part, and Euler mesh is used for other areas.

In the above studies, the deformation and failure of steel plates with a thickness of 1 to 10 mm were mainly studied. In the study of thick steel plates [18], the amount of explosive (0.2 kg) used in the study and the contact area (10 cm × 5 cm) between the explosive and the pipeline steel were relatively large, and no cross-cut analysis was performed on the pipe steel. The steel plate studied by Li [15] had prefabricated defects. At present, there is no research on the damage of a thick steel plate with a small charge and contact area. 

**Figure 1:**
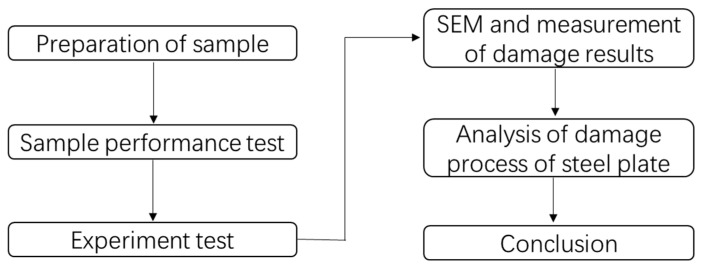
Flowchart regarding the overall study.

In this study, three steel plates with a thickness of 17 mm were selected to conduct an experimental study on the contact explosion of explosives. The microstructures of the fractures and fragments were analyzed by SEM, and numerical simulations were carried out to restore the experiment. Through the research, the influence law of the different equivalents of explosives and the contact area on the steel plate and the damage mode of the thick steel plate when the explosive is in contact with the explosion are obtained, and the reliability of the numerical simulation is verified according to the experimental results. Finally, through the analysis of numerical simulation results and experimental results, the damage criterion of the steel plate under contact explosion is obtained. The flowchart regarding the overall study is shown in Figure 1. The research results can provide data support and a theoretical basis for theoretical and protection research on steel.

## 2. Experimental Investigation

### 2.1. Test Specimens

**Figure 2:**
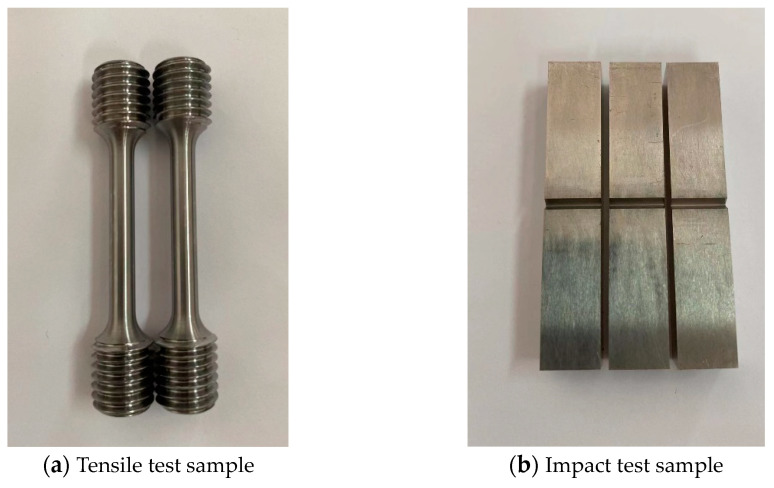
Test sample of steel plate.

The test specimens are three steel plates with the size 400 mm × 300 mm × 17 mm. The steel plate density is 7.83 × 10^3^ kg/m^3^, and the grade of steel is X80 steel. The steel plate is cut from a pipe with a diameter of 1219 mm, so the steel plate has a small curvature. Before the start of the experiment, the mechanical properties of the steel plates were tested, and two sets of tensile tests and three sets of impact tests were carried out on the five steel samples (see Figure 2). The results of the samples are shown in Table 1 and Table 2. The mechanical properties of the steel plate can be obtained by averaging the experimental results. The tensile strength is 651.5 MPa, the specified plastic elongation strength (R_P_0.2) is 544 MPa, the elongation at break is 26%, the area shrinkage is 77.5%, and the impact toughness is 317 J/cm^2^.

### 2.2. Experiment Setup and Measurement

In the experiment, 20 g, 50 g, and 100 g charges of TNT were selected. The specific parameters of the TNT are shown in Table 3.

**Figure 3:**
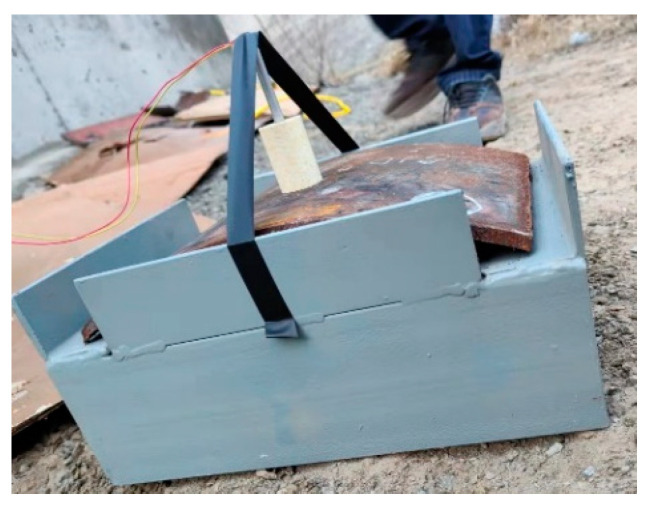
Layout of the experiment.

During the experiment, the steel plate was placed in a steel frame (Figure 3). The steel frame is made of a steel plate, and the interior of the steel frame is empty. During the experiment, the length of the contact position between the steel plate edge and the steel frame is 5 cm. At the beginning of the experiment, the steel plate was placed on the steel frame, and the safety operator placed the explosive in the center of the steel plate and fixed it. Finally, the safety operator detonated the explosive, and the experimental process was over. 

### 2.3. Comparison between Steel Plate Experiment and Large-Scale Experiment 

**Figure 4:**
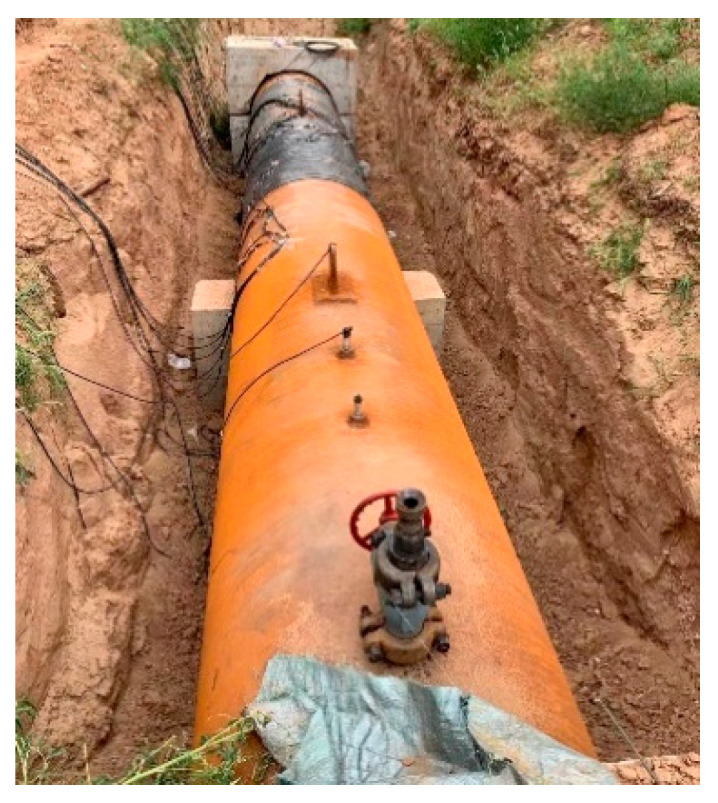
Experimental layout of large-size pipeline.

To examine the influence of the size effect and boundary conditions on the experimental results, the experimental comparison between the steel plate and the large-size pipeline was carried out before the beginning of the experiment. The experimental conditions of the steel plate were consistent with this study, and the large-scale experiments were verified using pipeline experiments. The diameter of the pipeline was 1219 mm, the wall thickness was the same as the thickness of the steel plate, and the length was 10 m. The two ends of the pipeline were fixed with concrete piers, and the explosive was placed on the upper part of the pipeline. The experimental layout of large-size pipeline is shown in Figure 4.

**Figure 5:**
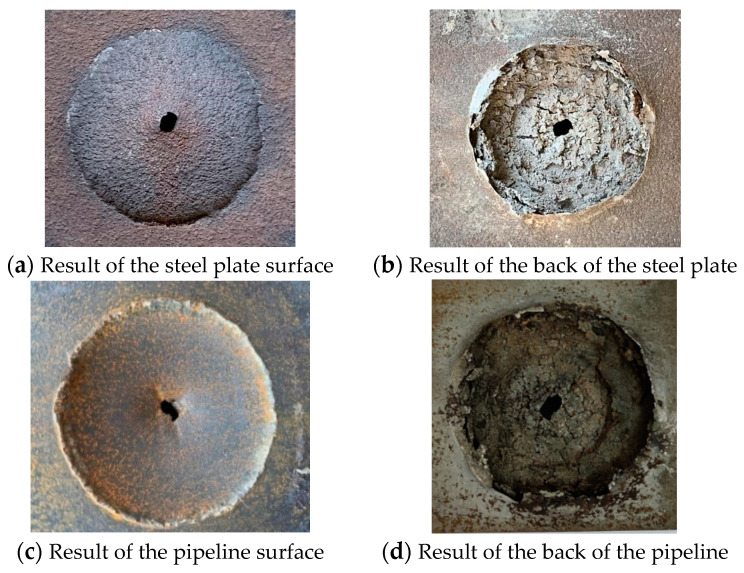
Experimental results of the steel plate and large-size pipeline.

The contact explosion experiments of the pipeline and the steel plate were carried out using the same energy condensed phase explosives. The experimental results are shown in Figure 5. Both the steel plate and the large-size pipe form a circular crater and a circular spall, and the center of the crater is perforated. The experimental results on the back of the pipeline were obtained after cutting the pipe. It can be seen that both the steel plate and the steel plate have oval perforations, large circular craters, and large circular spalls.

The specific damage parameters of the steel plate and the pipeline test are shown in Table 4. It is visible that the specific size difference between the steel plate and the pipeline is 1 mm, and the error is within an acceptable range. It can be judged that the results of the steel plate experiments in this study are similar to those of the large-scale experiments, and the effects of size effect and boundary effect on this study can be ignored.

## 3. Experimental Results and Discussion

### 3.1. Experimental Results

**Figure 6:**
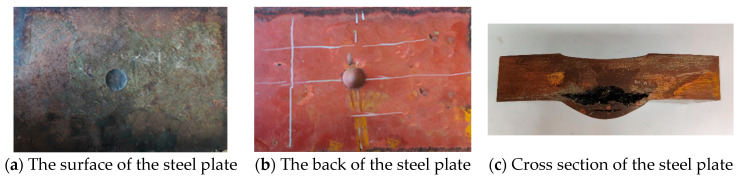
Experimental results for the steel plate when the TNT charge is 20 g.

Figure 6 shows the experimental results when the TNT charge of 20 g contacts the steel plate. A 31 mm circular crater is formed on the surface of the steel plate, the depth of the crater is 2 mm, a 31 mm conical protrusion is formed on the back, and the height of the protrusion is 7 mm. After cutting the steel plate laterally along the center of the crater, the damaged result of the cross-section of the steel plate was visible and is shown in Figure 6c. There are two cracks in the cross section of the steel plate. The larger crack has a length of 31 mm and a maximum width of 7 mm, and the smaller crack has a length of 11 mm and a maximum width of 0.5 mm. As shown in Figure 6c, there is an opening in the lower part of the cross section, while the upper part is relatively complete, and the thickness of the relatively complete part is defined as the remaining thickness. The remaining thickness of the steel plate is 10 mm. It can also be seen that the location of the crack is close to the back of the steel plate.

**Figure 7:**
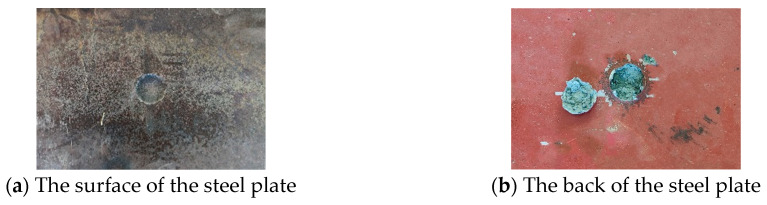

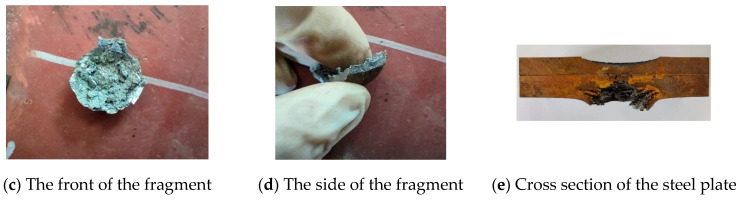
Experimental results for the steel plate when the TNT charge is 50 g.

Figure 7 shows the experimental results when the TNT charge of 50 g contacts the steel plate. A 34 mm circular crater is formed on the surface of the steel plate, the depth of the crater is 5 mm, a 29 mm conical protrusion is formed on the back, and the height of the protrusion is 8 mm. Fragments fall off on the back of the steel plate (Figure 7c,d). The shape of the fragment is close to hemispherical, with a protrusion at the inner center and a pit at the inner edge. There are silver fracture marks on the side of the fragment (Figure 7d), and the thickness at this position is 2 mm. 

The cross section of the steel plate is shown in Figure 7e. It can be seen that the cross section near the surface of the steel plate is intact, and the cross section near the back of the steel plate is severely damaged. The damage range is the largest at the lower surface of the cross section (near the back of the steel plate), and, as the distance between the cross section and the lower surface of the steel plate increases, the damage range of the steel plate decreases. There are three obvious cracks on the edge of the damage. The minimum distance between the damage position of the steel plate and the surface of the steel plate is 5 mm, of which there are small cracks of 3 mm, and the distance without obvious cracks is 2 mm. It can be concluded that when the TNT charge is 50 g, the remaining thickness of the steel plate is 2 mm.

**Figure 8:**
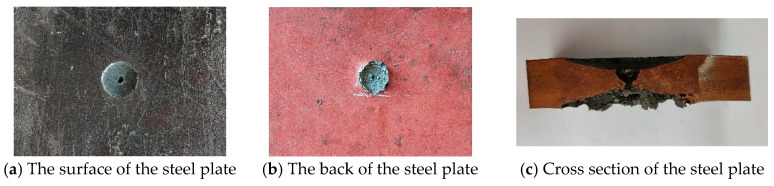
Experimental results for the steel plate when the TNT charge is 100 g.

Figure 8 shows the experimental results when the TNT charge of 100 g contacts the steel plate. A 42 mm circular crater is formed on the surface of the steel plate; the depth of the crater is 5 mm, and the diameter of the hole in the center of the crater is 8.5 mm. A 45 mm conical protrusion is formed on the back. A 45 mm circular spall is formed on the back of the steel plate, and a 3 mm diameter circular hole appears in the center of the spall.

The cross section of the steel plate is shown in Figure 8c. The area near the surface of the cross section is relatively intact, and only a hemispherical defect with a depth of about 4 mm is formed at the center. There is severe damage in the cross section near the back of the steel plate. Severe damage occurred in the area near the back of the steel plate of the cross section. From the cross section, the damage near the lower surface is similar to the triangle. 

**Figure 9:**
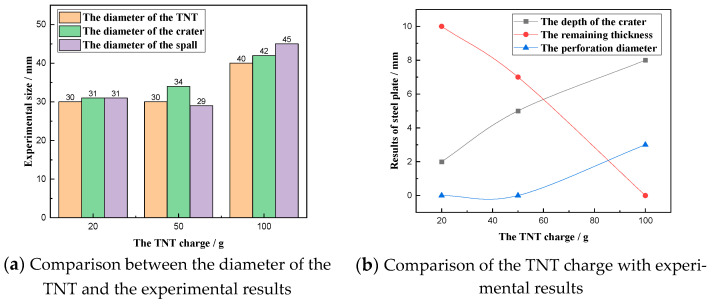
Relationship between experimental results and the TNT charge.

The comparison diagram shown in Figure 9 can be obtained by sorting out the experimental results. It can be seen from Figure 9a that the diameter of the explosive is close to the diameter of the crater on the surface of the steel plate. Furthermore, it can be found that the diameter of the crater on the surface of the steel plate is larger than that of the explosive in the three experiments. The diameter of the spall on the back of the steel plate is smaller than the diameter of the explosive when the TNT charge is 50 g. However, under the action of a TNT charge of 20 g and 100 g, the diameter of the back spall or protrusion is larger than that of the explosive. 

When the TNT charge increases from 20 g to 50 g, the equivalent rises by 150%, and the explosive diameter does not change. In this situation, the diameter of the crater on the surface of the steel plate increased by only 9.6%, and the diameter of the pit on the back of the steel plate decreased by 3.2%. When the TNT charge increases from 50 g to 100 g, the equivalent increases by 100%, the explosive diameter increases from 30 mm to 40 mm, and the diameter increases by 33.3%. At this time, the diameter of the crater on the surface of the steel plate increased by 23.5%, and the diameter of the spall on the back of the steel plate increased by 55.2%. From the above data, it can be seen that the increase of the TNT charge is less than the effect of the increase of the explosive diameter on the crater diameter on the steel plate. It can be concluded that when the explosive contacts the steel plate for an explosion, the contact area between the explosive and the steel plate has a significant influence on the size of the steel plate crater. 

Figure 9b shows the relationship between the failure parameters of the steel plate and the explosive charge. It can be seen from the figure that with the increase of the explosive charge, the depth of the crater on the surface of the steel plate increases, and the remaining thickness of the steel plate decreases. The main reason is that when the TNT charge increases, the impulse acting on the surface of the steel plate increases. For the perforation diameter of the steel plate, it can be seen that the steel plate will not be perforated when the TNT charge is small. When the TNT charge is 100 g, the steel plate is perforated. This phenomenon happens because the TNT charge of 100 g produces an explosion impulse that reaches the strength to perforate the steel plate.

From the above discussion, it is apparent that the diameter of the explosive mainly affects the size of the crater on the surface of the steel plate. In contrast, the change of the TNT charge will affect the depth of the crater on the surface of the steel plate, the remaining thickness of the steel plate, and the perforation diameter of the steel plate. In other words, when a local contact explosion occurs, the diameter of the contact surface between the explosive and the steel plate affects the damage range of the steel plate, and the TNT charge determines the damage degree of the steel plate.

### 3.2. Analysis of Damage Position of Steel Plate by SEM 

To study the damage process of the steel plate when the explosive makes contact with the explosion, the SEM image analysis was carried out on the perforation of the steel plate, the center, and the edge of the fragment. The SEM used in the experiment is Zeiss EVO25.

**Figure 10:**
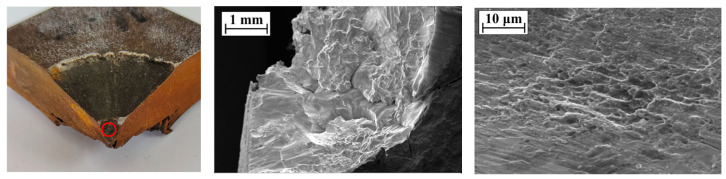
SEM image of the perforation of the steel plate (The red circle is the SEM test position).

**Figure 11:**
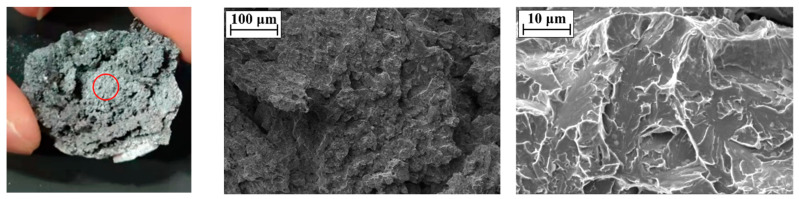
SEM image of the center of the steel plate fragment (The red circle is the SEM test position).

**Figure 12:**
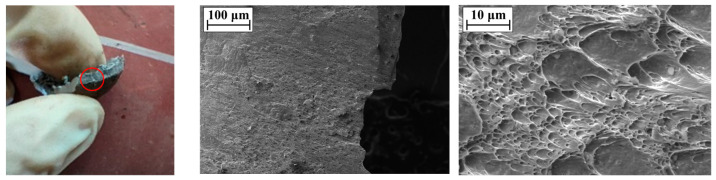
SEM image at the edge of the steel plate fragment (The red circle is the SEM test position).

The SEM image of the perforation of the steel plate is shown in Figure 10. The microstructure of the perforation surface of the steel plate is dominated by dimples, from which it can be inferred that the fracture mode of the perforation position is a ductile fracture. The SEM image of the center of the steel plate fragment is shown in Figure 11. There are a large number of torn edges in the microstructure on the surface of the center of the fragment, so it can be considered that the fracture mode of the center of the fragment is a quasi-cleavage fracture; that is, both ductile and brittle fractures exist at the center during the formation of the fragment. The SEM image of the edge of the steel plate fragment is shown in Figure 12. There are a large number of dimples in the microstructure on the surface of the edge of the fragment, thus it can be considered that the fracture mode of the edge of the fragment is a ductile fracture.

From the above discussion of SEM images at different positions of perforations and fragments, it can be seen that the fracture modes of the perforation of the steel plate and the edge of the fragments are similar; both of these are ductile fractures, while the center of the fragments has both ductile fractures and brittle fractures. From the experimental results of Section 3.1, it can be seen that the steel plate cracks exist in all the experiments, and the spall edges on the back of the steel plate and perforation will finally be formed. That is, when the steel plate is in contact with the explosive, the fracture mode of the crack forming process of the steel plate is a quasi-cleavage fracture, and the process of forming spall edges on the back of the steel plate and cracks is a ductile fracture.

### 3.3. Failure Mode of Steel Plate in Contact with Explosive 

**Figure 13:**
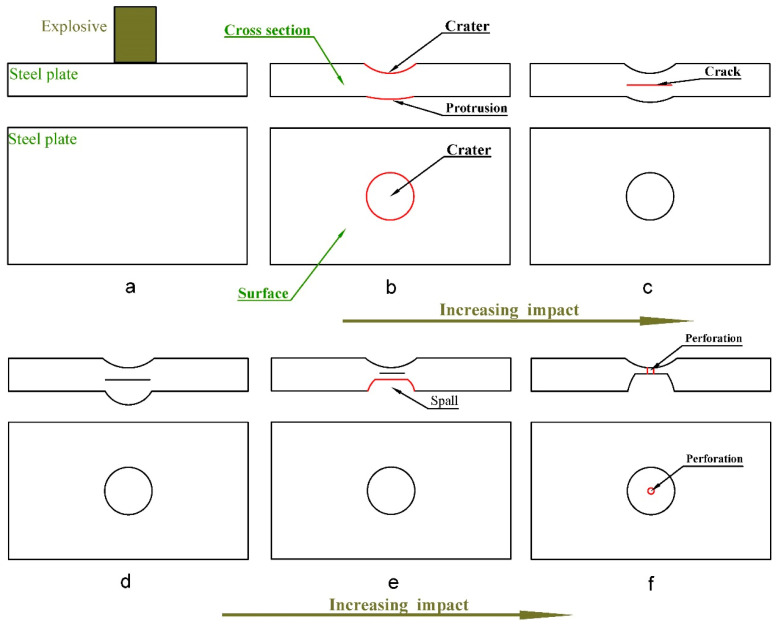
The changing state of the steel plate when the shock wave strength increases. ((**a**) initial state. (**b**) Slight bending without cracks. (**c**) slight bending with cracks. (**d**) damage mode (I). (**e**) damage mode (Ⅱ). (**f**) damage mode (Ⅲ)).

From the above experimental results, it can be seen that the damage of the steel plate is quite different under the different TNT charges. The main reason for this is the increase in the impulse caused by the increase of the TNT charge. After the explosive explodes, the generated shock wave will first act on the contact position between the steel plate and the explosive. The strong shock wave causes craters on the surface of the steel plate and the back of the steel plate protrusions (Figure 13b). When the shock wave acts on the steel plate, it will continue to propagate in the direction of the thickness of the steel plate. When the shock wave propagates to the interface between the steel plate and the air, and because of the difference in impedance between the air and the steel, the shock wave will propagate in the opposite direction in the form of a reflected wave. When the reflected wave intensity is large enough, cracks will be formed inside the steel plate through a quasi-cleavage fracture (Figure 13c). This is the reason for the result when the TNT charge of 20 g acts on the steel plate.

When a crack is formed, the steel plate can be divided into two parts along with the crack position, the upper part from the surface of the steel plate to the crack position and the lower part from the crack to the back of the steel plate. Subsequent shock waves will continue to compress the lower part of the plate, resulting in a larger protrusion on the back of the plate (Figure 13d). When the subsequent shock wave strength is large enough, the lower part of the steel plate will form fragments through a ductile fracture along with the crack position (Figure 13e). This is the reason for the damage of the steel plate when the TNT charge of 50 g acts on the steel plate.

Due to the existence of cracks, the thickness of the upper part of the steel plate is reduced compared to the original steel plate, so that under the action of the subsequent shock wave, the upper part of the steel plate continues to dent. At the same time, the reflected wave caused by the shock wave will also cause cracks in the upper part of the steel plate. Figure 7e is caused by this phenomenon. If the shock wave strength is sufficiently large, the steel plate will be punctured, causing piercing damage to the steel plate (Figure 13f). This is the reason for the result when the TNT charge of 100 g acts on the steel plate.

It can be seen from Figure 13 that the steel plate will go through five different changing processes when the explosive explodes. However, in the actual process, slight bending can also lead to cracks. Therefore, when considering the damage mode of the steel plate, the process where the steel plate does not have cracks (Figure 13b) can be ignored. The following can be obtained for the failure mode of the steel plate when the different TNT charges make contact with the explosion: (I) A crater on the surface of the steel plate and a protrusion on the back, and internal cracks in the steel plate. (II) A crater on the surface and a spall on the back of the steel plate. (III) A crater on the surface and a spall on the back of the steel plate, and the center of the crater is perforated.

Damage mode (I), after the explosion shock wave acts on the steel plate, the surface of the steel plate will produce a crater similar in shape to the contact part between the steel plate and the explosive, and the size of the crater is close to the size of the contact part. A conical protrusion is formed on the back of the steel plate, and the size of the bottom surface of the cone is close to the size of the contact part. Cracks appear inside the steel plate, the size of the crack is close to the size of the contact part, and the position of the crack is close to the back of the steel plate.

Damage mode (Ⅱ), after the explosion shock wave acts on the steel plate, the damage of the steel plate surface is similar to the failure mode (I). The back of the steel plate has a spall due to failing of the fragments, and the size of the spall is close to the size of the contact part between the steel plate and the explosive. Compared with the original thickness of the steel plate, the thickness of the contact position between the steel plate and the explosive becomes thinner, and the thickness of the steel plate at the center of the contact position is the thinnest, and the thinning trend of the thickness decreases from the center of the contact position to the edge.

Damage mode (Ⅲ), after the explosion shock wave acts on the steel plate, the damage of the steel plate surface and the back of the plate surface are similar to the damage mode (Ⅱ). Different from other damage modes, there are perforations in the crater of the steel plate, and the size of the perforations is determined by the strength of the shock wave.

## 4. Numerical Model

Compared with experimental research, numerical simulation has the advantages of high efficiency, safety, and low cost. Numerical simulations verified by experimental data can be used as predictions and supplements to experiments, thereby reducing the environmental pollution and the waste of resources caused by invalid experiments. 

### 4.1. Description of the Model

**Figure 14:**
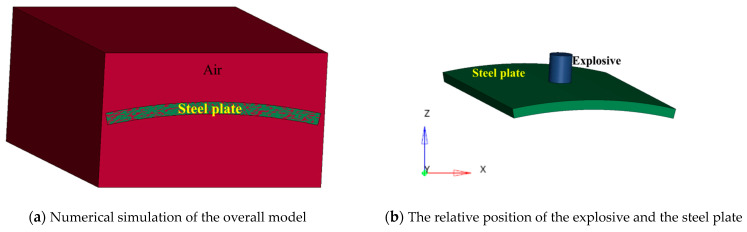
Numerical simulation model.

The numerical simulation model is shown in Figure 14, and a full-scale model is used for modeling. Compared with the experiment, the numerical simulation model includes a steel plate, an explosive, and air. The steel frame supporting the steel plate is omitted; the main reason is that the duration of the explosion is extremely short, and the relative sliding between the steel plate and the steel frame can be ignored. Omitting the steel frame does not affect the results of the numerical simulation, and, at the same time, the model can be simplified, and the calculation efficiency can be improved. In the numerical simulation, the steel plate and the explosive are the same size as in the experiment. The size of the air model is 400 mm × 310 mm × 200 mm. The explosive and the air are set to the Euler grid, the grid element adopts a multi-material ALE algorithm, the steel plate is set to the Lagrangian grid, and the fluid–solid coupling algorithm is used between the steel plate and the air and the explosive. The mesh type used is hexahedral mesh. The boundary conditions of the air and the steel plate are set to the non-reflecting boundary.

### 4.2. Material Models

The material model of the explosive [30] is selected to be MAT_HIGH_EXPLOSIVE_BURN, and the equation of state is the JWL equation.

(1) $$p=A\left(1-\frac{\omega }{R_{1}V}\right)e^{-R_{1}V}+B\left(1-\frac{\omega }{R_{2}V}\right)e^{-R_{2}V}+\frac{\omega E}{V}$$

where p is the pressure of the explosive (Pa), V is the relative volume of detonation products, E is the internal energy per unit volume of the explosive (J/m^3^), and A, B, R_1_, R_2_, and ω are the parameters of the equation of state. In this study, the explosive density is 1.63 × 10^3^ kg/m^3^; detonation velocity is 6.93 × 10^3^ m/s, and the other parameters are as follows, A = 3.71 × 10^11^ Pa, B = 7.43 × 10^9^ Pa, E = 7.0 × 10^9^ J/m^3^, R_1_ = 4.15, R_2_ = 0.95, ω = 0.3.

The material model of the steel plate is MAT_JOHNSON_COOK [31,32], which is fit for modeling the strength behavior of materials subjected to the large strain, high strain rate, and strength behavior at a high temperature. The state equation is EOS_GRUNEISEN, and the model function is

(2) $$\sigma_{y}=\left(A+B{\bar{\varepsilon }}^{p^{n}}\right)\left(1+CI_{n}{\dot{\varepsilon }}^{*}\right)\left(1-T^{{*}^{m}}\right)$$

A, B, C, and m are constants; $\sigma_{y}$ is the flow stress, MPa; ${\bar{\varepsilon }}^{p}$ is the effective plastic strain, MPa; ${\dot{\varepsilon }}^{*}$ is the effective total strain rate; $T^{*}$ is the homologous temperature, $T^{*}=\frac{T-T_{room}}{T_{melt}-T_{room}}$,$T_{room}$ is the room temperature, and $T_{melt}$ is the melt temperature, K. In the numerical simulation of this paper, the material parameter is X80 steel and the density ρ = 7.83 × 10^3^ kg/m^3^, Young’s modulus E = 2.1 × 10^11^ Pa, Shear modulus G = 0.77 × 10^11^ Pa, and the other parameters A = 545 MPa, B = 1658.4 MPa, C = 0.0937, m = 1.03, n = 0.95582. The melt temperature is T_melt_ = 1793 K, and the room temperature is T_room_ = 294 K.

The material model of air [33] is MAT_NULL, and the state equation is EOS_LINEAR_POLYNOMIAL,

(3) $$P=C_{0}+C_{1}\mu +C_{2}\mu^{2}+C_{3}\mu^{3}+\left(C_{4}+C_{5}\mu +C_{6}\mu^{2}\right)E$$

(4) $$\mu =\frac{\rho }{\rho_{0}}-1$$

P is the air pressure (Pa); ρ is the density of air ($kg/m^{3}$); $\rho_{0}$ is the initial air density $\left(kg/m^{3}\right)$; E is the volume energy of the material ($J/m^{3})$; $C_{0}–C_{6}$ are the state parameters of the equation. The air parameter in the paper is ρ = 1.29 kg/m^3^; E = 0.25 × 10^9^ J/m^3^; C_0_ = −1.0 × 10^−6^; C_1_ = C_2_ = C_3_ = C_6_ = 0; C_4_ = C_5_ = 0.4.

### 4.3. Convergence Analysis

Appropriate mesh size can be obtained through convergence analysis, which can ensure the accuracy of numerical simulation and improve the calculation efficiency. During meshing, a variety of mesh sizes were used to verify the surface and cross-section of the steel plate. The mesh verification of the steel plate surface was first carried out. The mesh size of 1 mm, 2 mm, and 3 mm were verified, while the grid size of the cross section of the steel plate was set to 1 mm. The experimental results of the steel plate with the TNT charge of 100 g were selected for verification. The simulation results show that the surface crater size of the steel plate are 47 mm, 47 mm, and 47 mm, and the back spall sizes are 46 mm, 47 mm, and 47 mm, respectively. The crater sizes of the steel plates in the three grids are relatively close, but in the 1 mm and 3 mm grids, the steel plates are not perforated. The 2 mm grid produced a crack, so the surface grid size of the steel plate was selected to be 2 mm.

**Figure 15:**
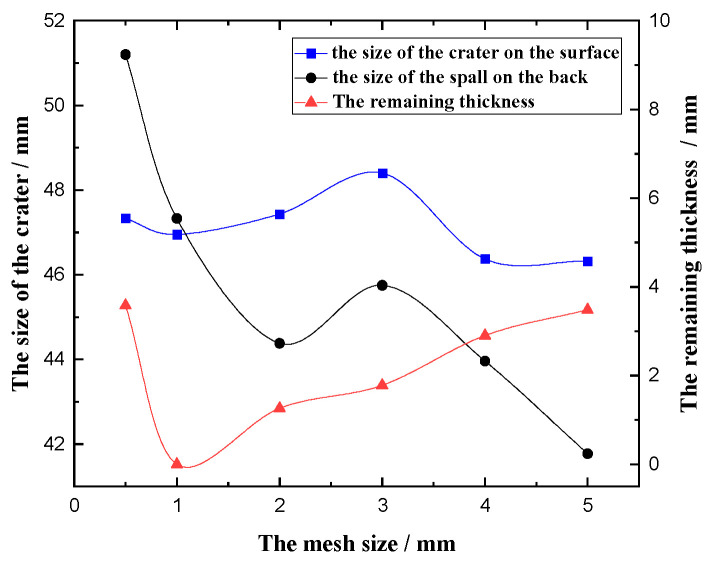
Numerical simulation results of different mesh sizes.

When verifying the grid size of the cross section of the steel plate, six different grid sizes of 0.5 mm, 1 mm, 2 mm, 3 mm, 4 mm, and 5 mm were selected. The simulation results are shown in Figure 15. It can be seen that different cross-section grid sizes have a greater influence on the size of the spall on the back of the steel plate. Meanwhile, with the increase in the mesh size, the remaining thickness of the steel plate first decreased and then increased. In the experimental results, the size of the crater on the surface of the steel plate is 42 mm, the size of the spall on the back is 45 mm, and the size of the spall on the back is larger than the size of the crater on the front. The steel plate is perforated; that is, the remaining thickness of the steel plate is 0. Compared with the experimental results, the size of the cross section of the steel plate is selected to be 1 mm.

## 5. Numerical Simulation Results 

### 5.1. Simulation Results When the TNT Charge Is 20 g 

**Figure 16:**
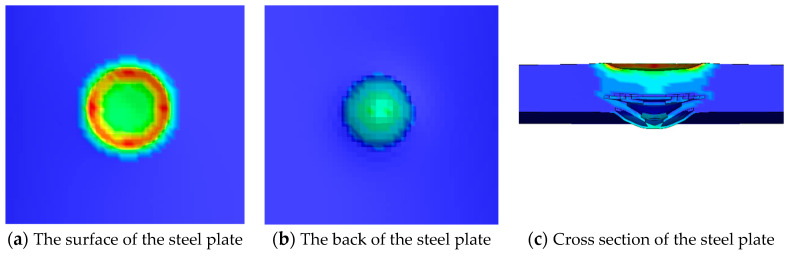
Numerical simulation results of the steel plate when the TNT charge is 20 g.

Figure 16 shows the numerical simulation results of the steel plate when the TNT charge of 20 g is in contact with the steel plate. The depth of the outline color represents the plastic deformation of the grid at this position, and the actual deformation can be seen in Figure 16c. A circular crater of 34 mm is formed on the surface of the steel plate, and the crater depth is 2 mm. A 30 mm conical protrusion is formed on the back of the steel plate, and the protrusion height is about 5 mm. Two cracks appeared on the cross-section of the steel plate, the larger crack length at the center of the cross-section was 34 mm, and the remaining thickness of the steel plate was 9 mm.

The comparison between the numerical simulation results and the experimental results is shown in Table 5. The numerical simulation results are close to the experimental results in terms of the diameter and depth of the surface crater, the diameter of the back protrusion, the diameter of the central crack in the cross section, the remaining thickness, and the error range is 0~10%. In terms of the protrusion height, the numerical simulation results are 2 mm smaller than the experimental results, and the error is 28.5%. The main reason is that the height of the protrusion is smaller, and the difference of 2 mm will also cause the error value to be larger. In general, the numerical simulation of the explosive charge of 20 g in contact with the steel plate is close to the experimental results in terms of the damage pattern of the steel plate and the damage parameters of the steel plate.

### 5.2. Simulation Results When the TNT Charge Is 50 g

**Figure 17:**
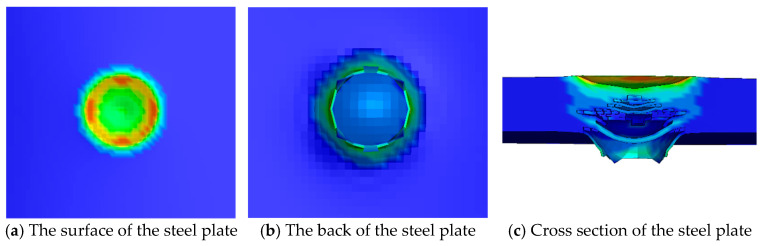
Numerical simulation results of the steel plate when the TNT charge is 50 g.

Figure 17 shows the numerical simulation results of the steel plate when the TNT charge of 50 g is in contact with the steel plate. The depth of the outline color represents the plastic deformation of the grid at this position, and the actual deformation can be seen in Figure 17c. A 36 mm circular crater is formed on the surface of the steel plate, and the crater depth is about 3 mm. A 30 mm circular spall is formed on the back of the steel plate, and the spall depth is about 11 mm. The area of the cross section near the surface is relatively intact, which is similar to the experimental results. Severe damage occurs in the area near the back of the steel plate in the cross section.

Compared with the cross section of the experimental steel plate (Figure 7e), the numerical simulation cross section (Figure 17c) shows that the lower part is not completely broken, adding a layer of curved bulges. The reason for this phenomenon is the difference between the broken form of the mesh in the numerical simulation and the experiment. The occurrence of failure in the experiment is a fracture, and the material at the fracture site remains. The damage in the numerical simulation is to delete the mesh when a certain preset condition is reached, which is equivalent to the reduction in part of the quality of the material, and this will lead to deviations in subsequent calculations. 

The comparison between the numerical simulation results and the experimental results is shown in Table 6. The numerical simulation results are close to the experimental results in terms of the diameter of the surface crater and back spall, with error ranges of 3.4% and 5.8%, respectively. In terms of the depth of the surface crater and back spall, the difference between the experiment and the numerical simulation is 2 mm and 3 mm, and the errors are 40% and 37.5%, respectively. The main reason for the large error between the two is that the crater depth is smaller than the crater diameter and other parameters. Even an error value of 3 mm will cause a large error. To sum up, there are some defects in the numerical simulation when the TNT charge is 50 g, but the diameter and shape of the crater of the steel plate are consistent with the experimental results, and the damage degree of the steel plate is slightly different from the experimental results. Therefore, the numerical simulation has a higher degree of reduction than in the experiment.

### 5.3. Simulation Results When the TNT Charge Is 100 g

**Figure 18:**
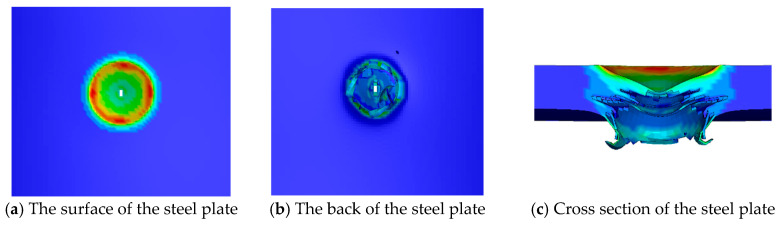
Numerical simulation results of the steel plate when the TNT charge is 100 g.

Figure 18 shows the numerical simulation results of the steel plate when the TNT charge of 100 g is in contact with the steel plate. The depth of the outline color represents the plastic deformation of the grid at this position, the white area in the center represents the position where a hole is formed, and the actual deformation can be seen in Figure 18c. A 47 mm circular crater is formed on the surface of the steel plate, and a square hole with a length of 4 mm and a width of 2 mm appears in the center of the crater, and the depth of the crater is about 9 mm. A 47 mm circular spall is formed on the back of the steel plate. The damaged area of the cross section near the upper surface is small. There is severe damage in the cross section near the lower surface, and there are some short cracks at the edge of the failure.

The comparison between numerical simulation results and experimental results is shown in Table 7. The numerical simulation results are close to the experimental results in all parameters, and the error range is 0–14.3%. Therefore, for the experimental results when the TNT charge is 100 g, the numerical simulation achieves an accurate reduction.

From the above discussion of the numerical simulation results, it can be seen that the numerical simulation will have some large errors for some small-sized features. However, it can achieve a very small error with the experimental results in many aspects, such as the diameter and shape of the crater of the steel plate, the diameter and shape of the protrusion, the perforation area, and the remaining thickness of the steel plate, and so on. Therefore, using the numerical simulation as the pre-experiment before the experiment means the results can be used as a reference for the experimental design.

## 6. The Criterion for Judging Damage Mode of Steel Plate

For contact explosions, the criteria for judging the failure mode of the steel plate should be studied. But there are no such studies on steel plates. In the research on contact explosions of concrete, McVay [34] first proposed an empirical formula of whether stress waves would cause spalling of reinforced concrete slabs. Based on McVay’s formula, Morishita [35,36] proposed the damage assessment formula of a concrete slab under a contact explosion. The formula is defined as follows (the unit is $cm\;/\;g^{\frac{1}{3}}$).

(5) $$\mathrm{No}\;\mathrm{spalling}:T/w^{\frac{1}{3}}>\;0.36$$

(6) $$\mathrm{Crater}\;\mathrm{and}\;\mathrm{spall}:\;0.2\;<\;T/w^{\frac{1}{3}}<\;0.36$$

(7) $$\mathrm{Occurrence}\;\mathrm{of}\;\mathrm{through}\;\mathrm{holes}:T/w^{\frac{1}{3}}<\;0.2$$

The T is the thickness of the reinforced concrete slab (cm) and the W is the equivalent amount of explosive in TNT (g). No spalling, Crater and spall, Occurrence of through holes correspond to Damage mode (I), Damage mode (Ⅱ) and Damage mode (III) in this study, respectively.

The number of explosives is considered in the above study, but at the same amount of explosive, if the radius of the bottom surface of the explosive is different, it will lead to the change of the impact strength acting on the steel plate, and the experimental results will be different. Therefore, the error of the method considering only the explosive charge will be relatively large. In the process of the contact explosion, the impact on the steel plate mainly comes from the impulse of the contact surface. Therefore, the error caused by the change in the shape of the explosive can be avoided by using the impulse of the contact surface instead of the explosive charge. Baum and Stanyukovich [37] proposed that when *l*(charge length) < 4.5*r* (bottom radius), the impulse at the bottom of the cylindrical charge is calculated as follows:

(8) $$I=\frac{8}{27}\left(\frac{4}{9}l-\frac{8}{81}\frac{l^{2}}{r^{2}}+\frac{16}{2187}\frac{l^{3}}{r^{3}}\right)\rho D\pi r^{2}$$

The *l* is the length of the explosive, *r* is the radius of the explosive, ρ is the density of the explosive, and *D* is the detonation velocity of the explosive. 

**Figure 19:**
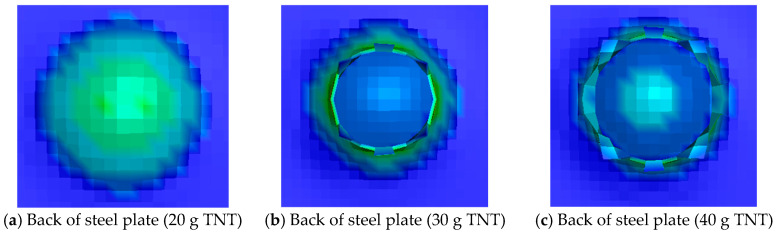

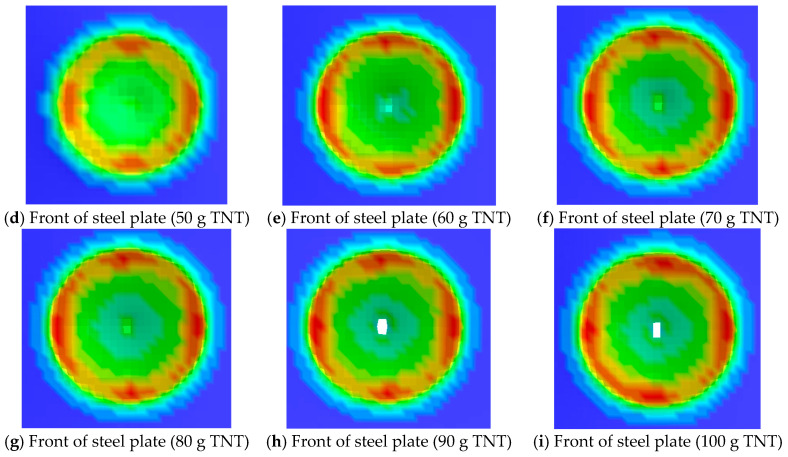
Damage results of steel plates under different explosive charges.

The reliability of the numerical simulation has been verified in the fourth part of this study. In order to obtain a more accurate relationship between impulse and steel plate failure, the numerical simulation study for the explosive charges of 30 g, 40 g, 60 g, 70 g, 80 g, and 90 g was added. The numerical simulation results are shown in Figure 19. It is apparent that when the explosive charge is 20 g, there is a protrusion on the back of the steel plate, that is, the steel plate is in damage mode (I). When the explosive charge is 30 g~80 g, the failure mode of the steel plate is similar, which belongs to the damage mode (Ⅱ). When the explosive charge is 90 g and 100 g, the steel plate is perforated, which is the damage mode (III) of the steel plate.

**Figure 20:**
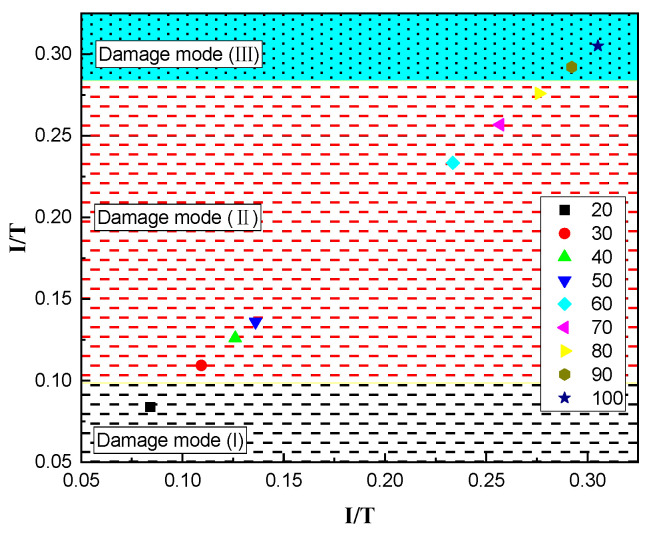
The corresponding relationship between the damage parameter I/T and the damage mode of the steel plate.

The data in Table 8 was obtained by calculating the impulse of the contact surface by Formula (8). Referring to the form of Morishita’s formula, the ratio I/T of the bottom surface impulse I (g·s) to the thickness of the steel plate T (cm) is used as the damage parameter to judge the failure mode of the steel plate during the contact explosion. Figure 20 is the diagram showing the corresponding relationship between the damage parameter I/T and the damage mode of the steel plate. From this, the judgment criteria for the corresponding damage parameter I/T can be obtained as follows,

(9) $$\begin{array}{cc} \mathrm{Damage}\;\mathrm{mode}\;(I) & I/T\leq 0.084\;\sim \;0.109\\ \mathrm{Damage}\;\mathrm{mode}\;(\mathrm{II}) & 0.084\;\sim \;0.109\leq I/T\leq 0.276\;\sim \;0.292\\ \mathrm{Damage}\;\mathrm{mode}\;(\mathrm{III}) & I/T\geq 0.276\;\sim \;0.292 \end{array}$$

The impulse of the contact surface can be obtained by Formula (8), and then the damage parameter I/T of the steel plate can be obtained by calculation. Finally, the damage mode of the steel plate under the contact explosion can be predicted by comparing it with the criterion for judging the damage mode (Formula (9)).

## 7. Conclusions

In this study, the failure results of a steel plate under a contact explosion of explosives were obtained using an experimental study, and the numerical simulation was verified by the experimental results. The main results are as follows.

(1) With the increase in explosive charge, the damage degree of the steel plate will change greatly. When the explosive charge of 20 g acts on the steel plate, craters and protrusions appear on the steel plate, and internal cracks appear. When the explosive charge of 50 g acts on the steel plate, there are fragments and deep craters in the steel plate. When the explosive charge of 100 g acts on the steel plate, the steel plate is perforated.
(2) The change of the contact area between the explosive and the steel plate has a greater effect on the diameter of the crater on the surface of the steel plate than the increase in the explosive charge. The increase of the TNT charge will lead to an increase in the depth of the crater on the surface of the steel plate and a decrease in the remaining thickness of the steel plate.
(3) The fracture mode of the steel plate in the process of generating cracks is a quasi-cleavage fracture, and the process of generating craters and perforations in the steel plate is a ductile fracture. The perforation surface of the steel plate and the edge of the fragment are dominated by dimples, while a large number of torn edges are formed on the center of the fragment.
(4) There are three damage modes of the steel plate. (I) A crater on the surface of the steel plate and a protrusion on the back, and internal cracks in the steel plate. (II) A crater on the surface and a spall on the back of the steel plate. (III) A crater on the surface and a spall on the back of the steel plate and the center of the crater is perforated.
(5) The comparison between the numerical simulation results and the experimental results proves that it is feasible to use numerical simulations to study the explosion experiment of the explosive contacting the steel plate. The difference between the numerical simulation results and the experimental results is between 0 and 5 mm, and the error interval of most parameters is between 0 and 14.3%.
(6) Through the calculation of the relevant parameters of the explosive and the steel plate, the damage mode of the steel plate under the contact explosion can be obtained. After calculating the impulse of the contact surface between the explosive and the steel plate and dividing it by the thickness of the steel plate, the damage parameter of the steel plate can be obtained. By comparing it with the judgment criteria, the failure mode of the steel plate can be obtained.
(7) More research is needed on the explosion results of explosive contact objects, and the effects of explosives on different new materials and combinations of materials can be explored in the future.

## Figures and Tables

**Table 1:** Tensile test results.

Sample Number	Test Temperature/°C	Tensile Strength /MPa	Specified Plastic Elongation Strength (R_P_0.2)/MPa	Elongation at Break/%	Area Shrinkage/%
1	25	649	545	25.5	78
2		654	543	26.5	77

**Table 2:** Impact test results.

Sample Number	Test Temperature/°C	Sample Specification/mm	Impact Toughness/J/cm^2^
3	25	10 × 10 × 55	314
4			320
5			317

**Table 3:** The detailed parameters of the TNT.

TNT Number	Charge/g	Bottom Diameter of the TNT/mm	Height of the TNT/mm
1	20	30	17.3
2	50	30	43.2
3	100	40	48.5

**Table 4:** Experimental results of the steel plate and large-size pipeline.

	Charge/g	Diameter of Surface Craters/mm	Perforation Size/mm	Diameter of Back Spalls/mm
Steel plate	150	53	5	50
Large-size pipeline	150	52	4	50

**Table 5:** Comparison of numerical simulation and experiment when the TNT charge is 20 g.

	Numerical Simulation/mm	Experiment/mm	Error/%		Numerical Simulation/mm	Experiment/mm	Error/%
Diameter of the surface crater	34	31	9.6	Depth of the surface crater	2	2	0
Diameter of the back protrusion	30	31	3.2	Height of the protrusion	5	7	28.5
Crack length	34	31	9.6	Remaining thickness	9	10	10

**Table 6:** Comparison of numerical simulation and experiment when the TNT charge is 50 g.

	Numerical Simulation/mm	Experiment/mm	Error/%		Numerical Simulation/mm	Experiment/mm	Error/%
Diameter of the surface crater	36	34	5.8	Depth of the surface crater	3	5	40
Diameter of the back spall	30	29	3.4	Depth of the back spall	11	8	37.5

**Table 7:** Comparison of numerical simulation and experiment when the TNT charge is 100 g.

	Numerical Simulation/mm	Experiment/mm	Error/%		Numerical Simulation/mm	Experiment/mm	Error/%
Diameter of the surface crater	47	42	11.9	Distance from surface to perforation	9	9	0
Diameter of the back spall	47	45	11.1	Perforation area	8	7	14.3

**Table 8:** Parameters and impulses of explosive charge.

TNT Charge/g	Length/mm	Bottom Radius/mm	Impulse I/g·s	I/T/g·s/cm
20	17.3	15	0.14283	0.08402
30	25.9	15	0.18584	0.10932
40	34.5	15	0.21429	0.12605
50	43.2	15	0.23132	0.13607
60	29.1	20	0.39695	0.2335
70	34	20	0.43660	0.25682
80	38.8	20	0.46926	0.27604
90	43.7	20	0.49685	0.29226
100	48.5	20	0.51876	0.30515

## Data Availability

Data can be obtained from corresponding authors upon reasonable request.
